# An explainable approach to fake news detection using PSO and mutual information

**DOI:** 10.3389/frai.2026.1901946

**Published:** 2026-10-07

**Authors:** Harisu Abdullahi Shehu, Paul D. Teal, Jeanelle Frontin, Amr Morssy, Mona Krewel, W. Bastiaan Kleijn

**Affiliations:** 1 School of Engineering and Computer Science, Victoria University of Wellington, Wellington, New Zealand; 2 School of History, Philosophy, Political Science and International Relations, Victoria University of Wellington, Wellington, New Zealand

**Keywords:** disinformation detection, explainable AI, fake news detection, feature selection, mutual information, Particle Swarm Optimization (PSO)

## Abstract

**Introduction:**

Fake news poses serious risks to societies by spreading disinformation rapidly and undermining trust in media. Automated methods for detecting fake news therefore need to be explainable to ensure transparency and trustworthiness. However, existing state-of-the-art methods are black-box and computationally expensive, making it difficult for practitioners and policymakers to understand and adopt them in real-world scenarios.

**Method:**

Feature selection can significantly enhance model transparency. Evolutionary computation (EC) methods are adaptable and naturally suited for optimization tasks such as feature selection. Among EC methods, Particle Swarm Optimization (PSO) requires few hyperparameters and converges quickly to good solutions. Therefore, this paper proposes an explainable approach to fake news classification using PSO. As mutual information (MI) quantifies the dependency between features and labels, the proposed method incorporates MI in its fitness function to guide the feature selection process and improve performance by eliminating redundant features.

**Result:**

Experimental results on three benchmark datasets (BuzzFeed, ISOT, and TruthSeeker) show that the proposed method achieved higher accuracy (85.21% on BuzzFeed, 99.99% on ISOT, and 90.92% on TruthSeeker) than baseline and state-of-the-art methods. Importantly, the proposed method selected a small feature subset, which helps explain model decisions, while requiring significantly less computation time.

**Discussion:**

These findings demonstrate the potential of PSO-based explainable approaches for practical and trustworthy fake news detection.

## 1 Introduction

Social media has become a dominant platform for information dissemination, enabling rapid communication and global connectivity (Khan et al., 2022). However, it has also amplified the spread of disinformation ˘ false or misleading information intentionally designed to deceive (A¨ımeur et al., 2023).

The rapid spread of disinformation has emerged as one of the most pressing challenges of the digital age (Kozik et al., 2023). The ability to identify and mitigate disinformation is critically important, as it undermines democratic processes, distorts public understanding of health and science, and threatens social cohesion (McKay and Tenove, 2021). Its harmful effects span various domains. For instance, in politics, it can manipulate elections and polarize societies; in public health, it can promote dangerous misinformation about treatments and vaccines; and in the social sphere, it can incite violence and hatred. These far-reaching consequences necessitate the development of robust and reliable detection mechanisms.

Traditional machine learning approaches have demonstrated moderate success in disinformation detection, typically relying on handcrafted linguistic and statistical features (Khan et al., 2025). However, their performance can be highly dependent on the quality and relevance of the extracted features, which may limit their ability to generalize across different news domains (Guo et al., 2026). While state-of-the-art deep learning models have demonstrated strong performance in identifying disinformation (Amira et al., 2023; Sormeily et al., 2024; Zhang et al., 2026), they operate as black-box systems, lacking intrinsic explanations for their decisions. Additionally, deep learning models are data-hungry due to their reliance on large datasets, which increases training time and computational complexity. Their use of large numbers of features can also result in overly complex models that are difficult to interpret, further hindering their practical deployment in real-world scenarios where transparency and efficiency are paramount.

To address these challenges, the application of feature selection is pivotal, as it determines the features most pertinent to classification and excludes irrelevant or repetitive ones (Shehu et al., 2021a). This not only reduces the dimensionality of the problem but also enhances classification performance by minimizing search space and increasing model generalizability.

Feature selection methods can be broadly categorized into filter-based and wrapper-based approaches. While filter-based methods rely on statistical properties of features and are computationally efficient, wrapper-based approaches optimize classifier performance directly, often yielding better results (Xue et al., 2012b).

In feature selection, the dimensionality of the solution space increases exponentially as the feature count rises. Consequently, exhaustive exploration becomes computationally prohibitive, leading to the employment of stepwise approaches that utilize greedy algorithms, including forward stepwise addition and backward stepwise removal methods (Ahmad and Zhang, 2009; Shehu et al., 2025), to minimize feature dimensionality. Nevertheless, such methodologies suffer from becoming stuck in local optima (Xue et al., 2014).

Conversely, evolutionary computation (EC) techniques, such as Particle Swarm Optimization (PSO), offer a promising alternative due to their global search capabilities. PSO is particularly advantageous for feature selection as it is simple to implement, converges quickly, and maintains computational efficiency (Coello, 2011).

To further enhance PSO's performance, Mutual Information (MI) will be incorporated as a guiding metric. MI measures the dependency between features and class labels, helping to identify the most discriminative features while reducing redundancy (Morssy et al., 2023). Therefore, it is anticipated that by integrating MI into PSO, the search process will become more effective, leading to an optimal feature subset that improves classification accuracy and model explainability.

The main goal of this work is to develop an explainable disinformation detection method that combines MI and PSO to select optimal features while reducing computational complexity. The scientific significance of this work lies in its novel integration of MI and PSO to enhance the accuracy, explainability, and computational efficiency of disinformation detection, while addressing the key limitations of current state-of-the-art methods.

The main contributions of this work are as follows:

1. Introduce a feature selection method that combines MI with PSO to enhance disinformation detection performance by selecting the most informative features.
2. Demonstrate the explainability of the proposed approach by leveraging the selected features to enhance transparency in model decisions, addressing the black-box nature of conventional deep learning methods. This is further investigated through a comparison with LIME (Ribeiro et al., 2016) and SHAP (Lundberg and Lee, 2017) to assess the method's effectiveness relative to established explainable AI methods in identifying and interpreting key features influencing model predictions.
3. Improved computational efficiency through feature reduction, which decreases training and evaluation time while maintaining high classification accuracy.
4. Experimental results demonstrating the generalizability and effectiveness of the proposed method on three state-of-the-art fake news datasets, namely (i) BuzzFeed (Horne and Adali, 2017), (ii) ISOT (Bozkus, 2022), and (iii) TruthSeeker (Dadkhah et al., 2023), through comparisons with baseline models, state-of-the-art deep learning models (i.e., BERT: Devlin et al., 2019; RoBERTa: Liu et al., 2019; GPT-2: Brown et al., 2020), and similar disinformation detection studies.

The remainder of the paper is organized as follows: Section 2 provides background on feature selection and highlights recent studies on fake news detection using both state-of-the-art and explainable approaches. Section 3 introduces the proposed methodology, including the feature extraction technique, the Particle Swarm Optimization algorithm, and the proposed fitness function. This section also explains the use of mutual information for optimizing the fitness function to improve the performance of the proposed method. Section 4 presents the experimental work, including a description of the datasets used, parameter settings, an explanation of the baseline and state-of-the-art methods used for comparison, as well as the experimental results. Section 5 discusses the findings of the paper and suggests directions for future research, and finally, Section 6 concludes the paper.

## 2 Background

This section covers feature selection, highlights recent work on explainability, as well as state-of-the-art approaches to fake news detection.

### 2.1 Feature selection

Feature selection is a critical step in machine learning, aiming to reduce dimensionality by selecting the most relevant features from a given dataset (Guyon and Elisseeff, 2003). This process not only improves model performance but also enhances interpretability by eliminating redundant or irrelevant features. In disinformation classification, feature selection becomes essential, as understanding the rationale behind predictions is crucial for ensuring trust, transparency, and accountability (Zhou and Zafarani, 2020). Among various metaheuristic techniques, Particle Swarm Optimization (PSO) has gained considerable attention for feature selection due to its simplicity, fast convergence, and ability to effectively explore the solution space.

Inspired by the social behavior of birds and fish, PSO models candidate solutions as particles moving through a search space (Eberhart and Kennedy, 1995), adjusting their positions based on both individual experience and social influence (see Section 3.2). When applied to feature selection, PSO efficiently identifies a subset of features that optimally balances classification accuracy with a minimal feature set size. This optimization significantly reduces computational overhead, which is particularly important when dealing with the high-dimensional textual data, typical of most disinformation datasets, and enhances model interpretability by focusing on the most informative attributes. A detailed explanation of how PSO is implemented is provided in Section 3.

### 2.2 Related work

This section examines current approaches to fake news detection, from high-performing transformer-based models that often lack interpretability to recent explainable methods that face challenges with computational efficiency and real-world applicability.

#### 2.2.1 State-of-the-art approaches to fake news detection

Fake news detection has become a crucial task in combating disinformation on digital platforms. Research has shifted from traditional machine learning to deep learning and transformer-based architectures (Kumar et al., 2024), which leverage contextual understanding and pre-trained language models to outperform older methods.

A key breakthrough was the adoption of BERT (Bidirectional Encoder Representations from Transformers), widely adapted for fake news detection. For example, Kaliyar et al.'s FakeBERT achieved 98.90% accuracy on a balanced real/fake news dataset (Kaliyar et al., 2021). Similarly, Pavlov and Mirceva fine-tuned BERT and RoBERTa on COVID-19 disinformation using ConstraintAI's dataset, raising BERT's accuracy from 34.57% to 98.31% and RoBERTa's from 55.04% to 97.52% (Pavlov and Mirceva, 2022). While effective, these models require domain-specific fine-tuning to handle evolving contexts.

RoBERTa, a refined BERT variant, was applied by Angizeh et al. on the LIAR dataset, which includes short, nuanced statements labeled across six truthfulness categories. RoBERTa improved accuracy slightly over BERT (from 64% to 66%) (Angizeh and Keyvanpour, 2024), benefiting from design changes like removing the Next Sentence Prediction objective and using longer input sequences during pretraining (Liu et al., 2019). Other models such as XLNet (Kumar et al., 2021) and DeBERTa (He et al., 2021) improve on BERT by capturing bidirectional context in novel ways or disentangling content and positional representations in attention mechanisms.

Ensemble methods have also gained traction. Aklouche et al. combined BERT, RoBERTa, and DistilBERT [a lighter, faster BERT variant (Sanh et al., 2020)] using majority voting and averaging strategies, achieving 67.88% on LIAR and 99.94% on ISOT (Text + Titles), improving robustness but increasing inference time and resource demands (Aklouche et al., 2024).

Multimodal approaches represent another innovation, integrating text with images and social cues typical of platforms like Facebook and Instagram. Xu et al.'s MAGIC model combined BERT for text and ResNet50 for images in a graph-based framework, achieving 98.8% on Fakeddit (English) and 86.3% on a Chinese dataset (Jun-hao and Xu, 2024). However, these models face challenges with generalizability and training complexity. Similarly, FakeNED fused BERT-based text features with VGG-16 image embeddings, achieving 87.79% on Fakeddit while demonstrating resilience to adversarial attacks, though it was limited by domain dependence and lacked source credibility modeling (Della Sciucca et al., 2022).

Overall, BERT-based approaches consistently outperform classical, CNN, and LSTM models on benchmarks like ISOT, LIAR, and FakeNewsNet (Kozik et al., 2023). Yet issues remain with generalizing to new disinformation formats, adapting to low-resource settings, and resisting adversarial inputs. For instance, Mridha et al. categorized approaches into RNN-based, CNN-based, hybrid, and transformer-based models, observing that while transformer models typically deliver the best performance, they also come with higher training costs and struggle with domain shifts (Mridha et al., 2021).

The choice of benchmarks also influences the performance reported, e.g., while ISOT (Bozkus, 2022) is popular for its clean binary labels, LIAR's fine-grained truthfulness labels pose greater challenges and FakeNewsNet (Shu et al., 2020) adds social context (likes, shares), helping models account for propagation dynamics.

In summary, transformer-based models—especially BERT, RoBERTa, DeBERTa, XLNet etc. and their ensemble or multimodal extensions represent the current state-of-the-art in fake news detection. Despite strong performance on benchmark datasets, challenges around generalization, explainability, computational cost, and bias remain to be fully addressed. To address these, this study introduces an explainable fake news detection approach that leverages Particle Swarm Optimization (PSO) to select meaningful, context-rich textual features. By reducing reliance on sparse representations and enhancing feature relevance, the proposed method aims to address the challenges such as generalization, explainability, and computational cost faced by existing state-of-the-art models.

#### 2.2.2 Explainable approach to fake news detection

The rapid spread of misinformation on digital platforms has spurred significant research efforts to develop reliable fake news detection systems. Many users uncritically trust social media content, often without verifying its credibility, necessitating robust methods that leverage both textual and multimedia elements to enhance detection accuracy.

Hashmi et al. (2024) proposed a hybrid method combining a Convolutional Neural Network (CNN) and Long Short-Term Memory (LSTM) model enriched with FastText embedding to predict fake news using the WELFake, FakeNewsNet, and FakeNewsPrediction datasets. They performed preprocessing steps including the removal of HTML tags and special characters, followed by tokenization. The authors then extracted FastText embeddings, which were fed into the proposed hybrid model as well as into traditional machine learning algorithms such as Logistic Regression (LR), Support Vector Machines (SVM), Decision Trees (DT), and Random Forest (RF) along with several state-of-the-art models including transformer-based architectures such as XLNet, RoBERTa, and BERT. The proposed CNN-LSTM model outperformed the other methods, achieving up to 99% accuracy. To enhance explainability, the authors utilized Latent Dirichlet Allocation (LDA) and Local Interpretable Model-agnostic Explanations (LIME) to identify and interpret the key features influencing predictions. However, the proposed method is data-hungry due to its deep learning architecture, and since deep networks require large amount of training data, the efficacy of the method remains questionable when applied to small datasets.

Khan et al. (2022) developed a co-attention sub-network framework that leverages sentence-comment interactions to detect fake news on the GossipCop and PolitiFact datasets. The model jointly leverages user comments and news content to identify the top-k explainable, check-worthy user comments and sentences that contribute to detecting fake news. To evaluate its effectiveness, the performance of the proposed model was compared against baseline models such as CNN and HAN, as well as state-of-the-art approaches, including the Hierarchical Propagation Network with Social Attention and Bi-Directional LSTM (HPAB-LSTM) (Guo et al., 2018), and dEFEND (Shu et al., 2019). The proposed model outperformed state-of-the-art methods by 5.56% and baseline methods by 16.4%, demonstrating its superiority in capturing relevant textual and social context for fake news detection. However, this approach depends on the presence and reliability of user feedback, which may vary across platforms and thereby limit its generalizability and robustness in real-world scenarios.

(Rao Kondamudi and Ranjan Sahoo 2025) proposed an explainable fake news detection dataset by combining three different fake news datasets (i.e., GossipCop, PolitiFact, and Snopes). They applied preprocessing techniques such as stopword removal, tokenization, and re-sampling, followed by the extraction of TF-IDF features from the text. Four classifiers, i.e., LR, SVM, DT, and RF were then used to predict whether the news was real or fake. SHAP (Lundberg and Lee, 2017) was employed to explain the predictions made by the models. Among the classifiers, the RF model achieved the highest performance with an accuracy of 96%, outperforming the others, which achieved less than 91% accuracy. Importantly, the SHAP technique highlighted the most influential features for identifying fake news, offering valuable insights into the model's decision-making process. However, the proposed approach relied on TF-IDF features, which tend to produce high-dimensional sparse vectors, especially with large vocabularies, thereby increasing memory consumption and computational overhead.

Hashmi et al. (2024), Khan et al. (2022), and (Rao Kondamudi and Ranjan Sahoo 2025) proposed explainable methods for predicting fake news. However, these methods are either computationally expensive, lack real-world applicability, or require large amounts of data due to their reliance on deep neural networks. To address these limitations, this study proposes an explainable fake news detection approach using Particle Swarm Optimization (PSO) with techniques that extract contextual textual features rather than sparse TF-IDF vectors, thereby improving performance on both small-scale and large datasets. Furthermore,the method aims to enhance explainability by selecting the most frequent and informative features that contribute to the prediction of fake news.

## 3 Methodology

Figure 1 presents the overall workflow of the proposed approach. Initially, two sets of features are extracted from the data, after which the dataset is divided into training, validation, and testing sets. To prevent feature selection bias and ensure that the test data remain unseen during the feature selection process, only the training and validation sets are provided to the PSO algorithm. The proposed PSO-based feature selection method follows a wrapper-based approach, in which the K-nearest neighbors (KNN) classifier is used to evaluate the classification performance of each candidate feature subset. Specifically, the selected features represented by each particle are used to train the KNN classifier on the training set, and the resulting validation error is incorporated into the fitness function. The PSO algorithm is guided by a multi-objective fitness function that incorporates mutual information to identify the most relevant and informative features while maintaining computational efficiency. Subsequently, the test set is evaluated using only the selected features from the training set to provide an unbiased assessment of the proposed approach.

**Figure 1:**
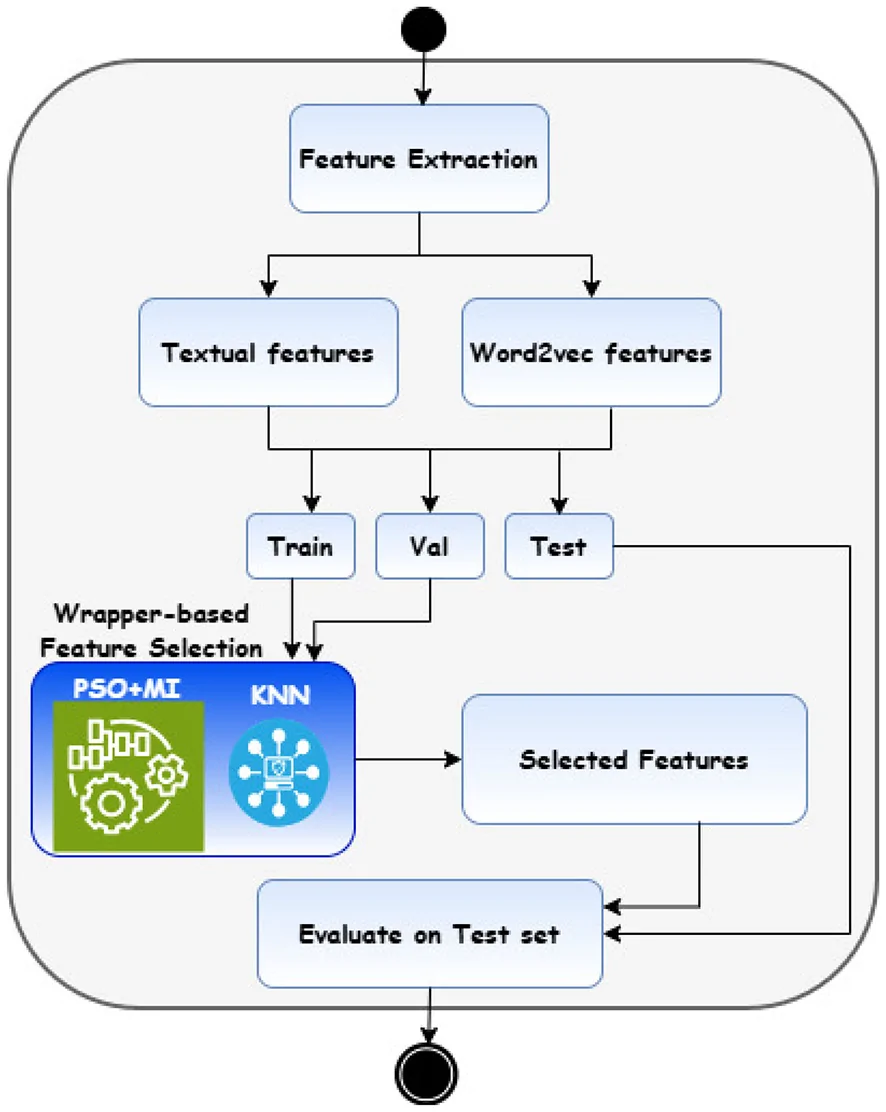
Overview of the proposed explainable fake news detection framework using PSO and mutual information.

The following subsections provide detailed descriptions of the components of the proposed approach.

### 3.1 Feature extraction

To enhance explainability, we extracted interpretable textual features in addition to the Word2Vec representations, because such features often capture meaningful linguistic and structural patterns that may be overlooked by dense vector representations such as TF-IDF, which tend to be less transparent and more difficult to interpret. A total of 16 features were extracted from both the BuzzFeed and ISOT datasets. Since the primary goal of fake news is often to manipulate or mislead, negative sentiments are expected to appear more frequently than positive ones, making sentiment-based features particularly useful for prediction. Additionally, features such as text length, word count, number of unique words, and the number of exclamation marks may contribute to detection, as fake news articles often use exaggerated or emotionally charged language, excessive punctuation, or unusual stylistic patterns to attract attention and provoke reactions.

For the Truthseeker dataset, a similar set of features was provided for use with traditional machine learning algorithms. In addition to the extracted features, we also incorporated Word2Vec and three additional linguistic features, i.e., positive terms, negative terms, and controversy-related terms that were not included in the initial feature set, bringing the total to 107 features.

Overall, these carefully selected textual features and Word2Vec representations are intended to improve both the predictive performance and explainability of the model by capturing linguistic and structural cues that distinguish fake news from legitimate content.

### 3.2 Particle Swarm Optimization

Particle Swarm Optimization (PSO) is a population-based metaheuristic inspired by the collective behavior of swarming agents (Eberhart and Kennedy, 1995). It employs a collection of potential solutions, referred to as “particles”, that navigate through a multi-dimensional solution space in pursuit of the global optimal point. Within a search domain of *D* dimensions, particle *i* is defined by its location vector **x**_*i*_ = (*x*_*i*1_, *x*_*i*2_, …, *x*_*iD*_) and corresponding velocity vector **v**_*i*_ = (*v*_*i*1_, *v*_*i*2_, …, *v*_*iD*_).

The movement of each particle is influenced by its own best-known position, **p**_*i*_ = (*p*_*i*1_, *p*_*i*2_, …, *p*_*iD*_), often referred to as *pbest*, and the single best-known position found by any particle in the entire swarm, **p**_*g*_ = (*p*_*g*1_, *p*_*g*2_, …, *p*_*gD*_), known as *gbest*.

At each time step *t*, the velocity of the *i*-th particle in the *d*-th dimension is adjusted according to its previous velocity, its cognitive experience (*pbest*), and its social experience (*gbest*). This update is formally described by the velocity update equation given by Equation 1.


$$\begin{array}{r} v_{id}\left(t+1\right)=w\cdot v_{id}\left(t\right)+c_{1}r_{1}*\left(p_{id}\left(t\right)-x_{id}\left(t\right)\right)\\ +c_{2}r_{2}*\left(p_{gd}\left(t\right)-x_{id}\left(t\right)\right) \end{array}$$ (1)

where the parameters *c*_1_ and *c*_2_ are acceleration coefficients that control the stochastic pull toward the *pbest* and *gbest* positions, respectively. The terms *r*_1_ and *r*_2_ are random values uniformly distributed within the range of [0, 1], introducing stochasticity into the search process.

Subsequently, the particle's position is updated using Equation 2.


$$\begin{array}{l} x_{id}^{t+1}=x_{id}^{t}+v_{id}^{t+1} \end{array}$$ (2)

where *d*∈*D* represents the *d*^*th*^ dimension, and *w* is the inertia weight, which moderates the influence of the prior velocity of the particle.

While the reliance on *gbest* and *pbest* often leads to rapid convergence, it also exposes a classic vulnerability of PSO, i.e., the risk of premature convergence. If the swarm converges on a local optimum too quickly, it may become trapped, failing to explore other promising regions of the search space (Coello, 2011; Chen et al., 2020). To address this, we propose enhancing the search guidance mechanism by employing a multi-objective fitness function, which incorporates Mutual Information (MI).

Since MI can quantify both the relevance of features to the target class and the redundancy between features, we hypothesize that an objective function that rewards high relevance and penalizes high redundancy can create a smoother fitness landscape, providing a more stable gradient for the swarm to follow. This helps prevent particles from oscillating or becoming fixated on solutions that are optimal only in terms of a single, potentially noisy, performance metric.

In essence, this approach steers the algorithm by redefining what constitutes a “good” solution, rather than by altering the core particle dynamics. This enhanced guidance mechanism is formalized in the fitness function described in Equation 4.

### 3.3 Fitness function

In PSO, the fitness function serves as the optimization criterion. It guides particles toward solutions that balance classification performance with feature selection quality.

A basic fitness function for this task might exclusively target the minimization of classification error, as defined by Equation 3:


$$\begin{array}{l} \text{ErrorRate}=\frac{\text{FP}+\text{FN}}{\text{TP}+\text{TN}+\text{FP}+\text{FN}} \end{array}$$ (3)

where TP, TN, FP, and FN represent true positives, true negatives, false positives, and false negatives, respectively. This approach prioritizes the enhancement of classification accuracy, while neglecting the objective of identifying a minimal yet highly relevant subset of features.

To address this, we formulated a multi-objective fitness function that balances three competing goals: maximizing classification accuracy, maximizing the information-theoretic value of the selected features via MI, and minimizing the size of the feature subset. The fitness function *F* to be minimized is a weighted sum of these three components, given by Equation 4.


$$\begin{array}{l} \begin{array}{c} F=\;\alpha \times \left(ErrorRate\right)+\left(\beta \times Î\right)\\ \;+\left(1-\alpha -\beta \right)\times \left(\frac{\#Features}{\#AllFeatures}\right) \end{array} \end{array}$$ (4)

where *#Features* represents the number of selected features, *ErrorRate* is presented in Equation 3, hyperparameter α controls the trade-off between the accuracy and number of features, and β controls the trade-off between the number of selected features and their relevance to the classification, as measured by the average mutual information (Î). A detailed description of how the MI is calculated is provided in Section 3.4.

The coefficients α and β are user-defined parameters within the range [0, 1] (with α+β ≤ 1) that control the trade-off between classification accuracy, information content, and feature set cardinality.

To optimize this objective function, we employ a continuous PSO variant. This choice is based on our preliminary experiments and findings in the literature (Xue et al., 2012a), which indicate that continuous PSO often yields higher accuracy compared to its binary counterpart for such tasks.

Algorithm 1 presents the procedure for selecting features from the disinformation datasets.

**Algorithm 1:** Continuous PSO algorithm for feature selection.

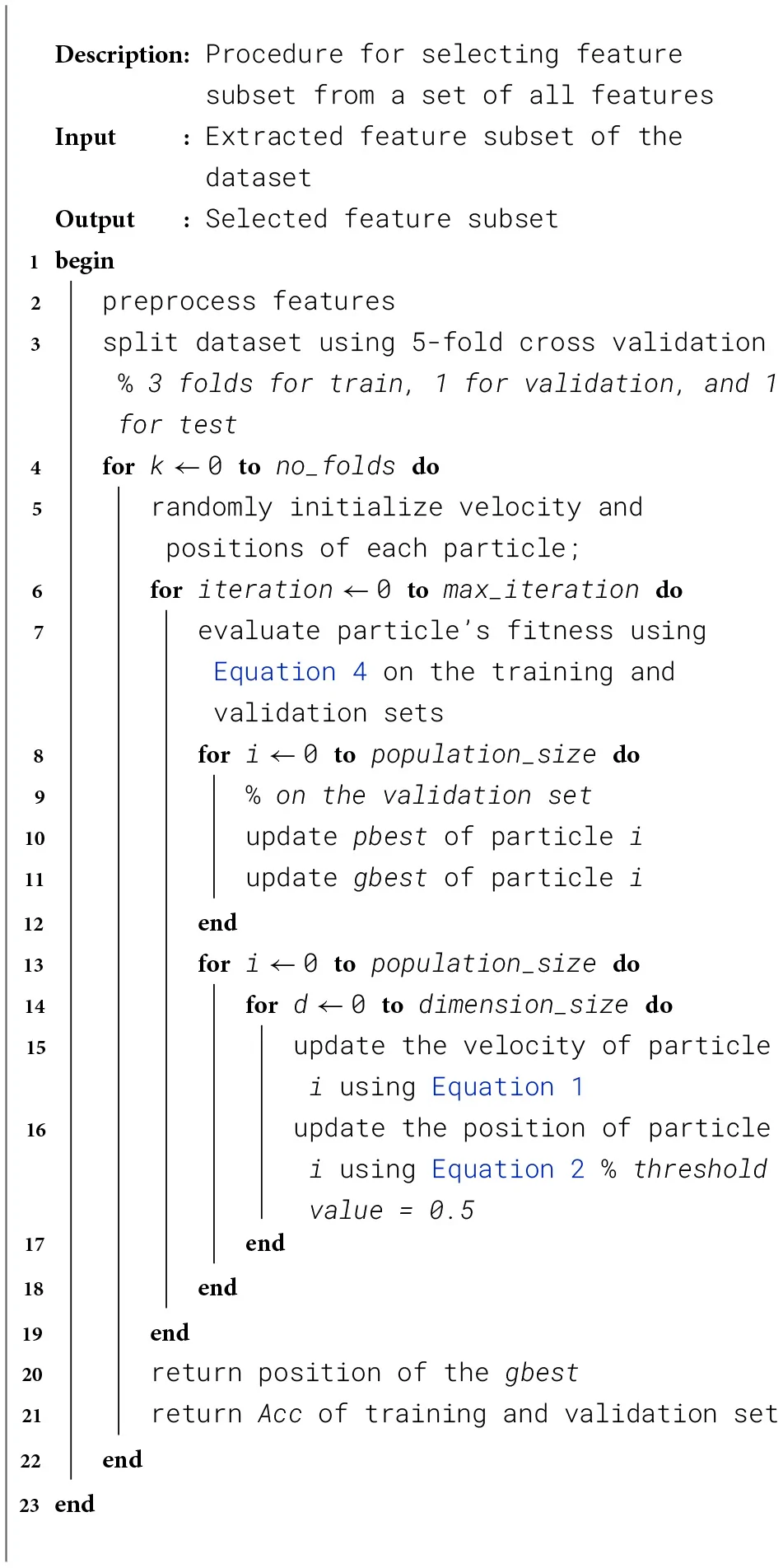

In the continuous PSO implementation, each particle's position vector represents a candidate feature subset, where each element *x*_*id*_ is constrained to the interval [0, 1]. The PSO search space is explicitly bounded to [0, 1]^*D*^ through the optimizer's boundary constraints. A feature *d* is included in the subset if its corresponding position value exceeds the threshold, i.e., *x*_*id*_>θ; otherwise, it is excluded. For this study, the threshold is set to θ = 0.5 (see Section 4.5). During fitness evaluation, if a particle selects no features, it is assigned an infinite fitness value, thereby treating the solution as infeasible and discouraging its selection. In the unlikely event that the final global-best position contains no selected features, the implementation falls back to the original feature set.

### 3.4 Mutual information

Here we introduce information theoretic concepts for the task of variable selection. The entropy *H*(*X*) of a discrete random variable *X* is a measure of the amount information in that variable and given by Equation 5:


$$\begin{array}{l} H\left(X\right)=-\underset{x\in X}{\sum }p\left(x\right)\log p\left(x\right), \end{array}$$ (5)

where *p*(*x*) denotes the probability mass function. This is a measure of the expected information associated with *X*. In the context of variable selection, we assume that *X* consists of multiple components *X* = {*X*_1_, *X*_2_, ⋯ , *X*_*N*_} and a corresponding label *Y* that we are interested in inferring. It is required to find the smallest subset *C*⊂*X* of components that allows the identification of the value *Y*. A natural way to approach this is to ask for the most informative subset of *X* about *Y*. The mutual information *I*(*X*; *Y*) for two discrete random variables *X* and *Y* measures the anticipated degree of information gain about one variable when the other is observed. This is given by Equation 6:


$$\begin{array}{l} I\left(X;Y\right)=H\left(X\right)-H\left(X|Y\right)\\ \;=\underset{x\in X}{\sum }p\left(x\right)\log p\left(x\right)-\left[\underset{x,y}{\sum }p\left(x,y\right)\log p\left(x|y\right)\right], \end{array}$$ (6)

where *H*(*X*|*Y*), and*p*(*x, y*) are the conditional entropy of X given Y, and joint probability mass of X and Y respectively.

In the case of a set of *M* variables, the chain rule for mutual information (see Equation 7) can be used:


$$\begin{array}{l} I\left(X_{1},X_{2},\cdots \;,X_{M};Y\right)=\\ \underset{i\to M}{\sum }I\left(X_{i};Y|X_{i-1},X_{i-2},\cdots \;,X_{1}\right). \end{array}$$ (7)

In practice, we typically do not have access to the true joint probability mass function of *X* and *Y*, but instead we are given samples *S* of pairs of *x, y* and as result we can only estimate the mutual information between them.

## 4 Experimental work

This section describes the experimental setup used to evaluate the proposed method, including the datasets employed, hardware specifications, evaluation protocols, and the baseline and state-of-the-art methods against which the proposed approach is compared.

### 4.1 Datasets

Three publicly available benchmark datasets are used to evaluate the proposed method, covering diverse news domains, scales, and media platforms. Table 1 provides a summary of the key characteristics of each dataset, with detailed descriptions presented in the following subsections.

**Table 1:** Summary of the benchmark datasets used in this study.

Dataset	Real	Fake	Total	Ratio (R:F)	Source
BuzzFeed	91	91	182	1:1	Facebook posts (2016 U.S. election)
ISOT	21,471	23,481	44,898	0.91:1	Reuters/PolitiFact & Wikipedia
TruthSeeker	68,930	65,268	134,198	1.06:1	Tweets (2009–2022)

#### 4.1.1 BuzzFeed dataset

The BuzzFeed News dataset (Horne and Adali, 2017) contains a full collection of Facebook posts published by nine news outlets during 2 weeks surrounding the 2016 U.S. election (September 19–23 and September 26–27). Each post, along with its associated article, was examined by a team of five BuzzFeed journalists. The fact-checking process produced two distinct files: one representing real news and the other representing fake news, with 91 entries in each, amounting to 182 records in total.

#### 4.1.2 ISOT dataset

The ISOT dataset (Bozkus, 2022) is a dataset of real and fake news. The truthful (real) articles in the dataset were acquired through web scraping from Reuters.com (a news platform), while the fake news pieces were gathered from various sources, encompassing questionable websites that had been identified by PolitiFact (a US-based fact-verification organization) and Wikipedia. Although the dataset encompasses articles across diverse subject areas, the predominant focus centers on political affairs and international news coverage. Overall, the ISOT dataset contains 21,471 real and 23,481 fake entries, i.e., a total of 44,898 entries across the two classes.

#### 4.1.3 Truthseeker dataset

The TruthSeeker dataset (Dadkhah et al., 2023) is one of the largest ground-truth tweet collections for real/fake content detection, covering the period from 2009 to 2022. Labels were sourced from PolitiFact and expert annotators, then refined through crowdsourcing on Amazon Mechanical Turk with multiple rounds of validation by highly skilled “Master Turkers.” The dataset supports both binary and multi-class classification schemes, with a primary focus on fake news detection in social media. Tweets were manually linked to relevant keywords extracted from real and fake news articles to ensure high data quality. Additional metadata includes user bot scores, credibility, and influence, enabling analyses of user behavior and tweet veracity. The dataset is available in multiple formats; however, this study uses its extracted feature set for machine learning, which comprises a total of 134,198 (68,930 real and 65,268 fake) entries.

### 4.2 Hardware specification

The experiments were conducted using an RTX A5000 GPU (NVIDIA) equipped with 24 GB memory and CUDA version 12.9.

### 4.3 Evaluation protocol

Five-fold cross-validation was used to assess generalization performance. For the baseline methods, in each run, four folds were used for training and the remaining fold for testing. For the proposed method and state-of-the-art methods, in each run, three folds were used for training, one fold for validation, and the remaining fold for testing. This primarily avoids feature selection bias and ensures fair model selection.

### 4.4 Compared methods

The proposed method is compared against baseline and state-of-the-art methods, as given below:

#### 4.4.1 Baseline methods

Three baseline methods, i.e., Decision Tree (DT), Random Forest (RF), and Logistic Regression (LR) were selected.

DT, RF, and LR are chosen for their ability to handle structured text-derived features effectively, with DT offering interpretability, RF providing robust ensemble learning, and LR delivering efficient, scalable learning suited to high-dimensional text data for fake news classification.

#### 4.4.2 State-of-the-art methods

Three state-of-the-art models, i.e., Bidirectional Encoder Representations from Transformers (BERT) (Devlin et al., 2019), A Robustly Optimized BERT Pretraining Approach (RoBERTa) (Liu et al., 2019), and Generative Pre-trained Transformer 2 (GPT2) (Brown et al., 2020) were selected.

BERT was chosen for its success in capturing semantic nuances critical to identifying fake news (Kaliyar et al., 2021).

RoBERTa was selected is selected for its improved training strategy that enables better detection of subtle linguistic cues and nuanced writing styles often found in fake news articles (Pavlov and Mirceva, 2022).

GPT2 was chosen for its ability to model and generate coherent, contextually appropriate text, which can help capture stylistic patterns and differentiate fake news from legitimate reporting (Gifu, 2023).

### 4.5 Parameter settings

The PSO implementation employed a swarm size of *P* = 30, with a maximum of T = 1, 000 iterations, cognitive and social coefficients set to *c*_1_ = *c*_2_ = 0.5, the seed to 42, the inertia weight to *w* = 0.9, the mutual information weight to β = 0.1, and the fitness function weight to α = 0.88. These parameters were determined using sensitivity analysis (see Supplementary material).

Since our objective is to simultaneously minimize feature dimensionality and optimize classification accuracy, we adopted a balancing factor of 0.5 to mediate the trade-off between model performance and feature count, following established conventions in prior studies (Miranda, 2018; Shehu et al., 2021b).

The KNN classifier used in the wrapper-based feature selection method was configured with five neighbors. The state-of-the-art methods we set up to use a *batch*_*size* (*bs*) = 256, *Epochs* = 3, *max*_*features* = 1000.

### 4.6 Experimental results

This section presents the results obtained by the proposed and compared methods. All statistical analyses in Sections 4.6.1–4.6.2 were based on the cross-validation results. For the Friedman and Iman-Davenport tests, each cross-validation fold was treated as a block, with the performance of each method within the same fold forming the corresponding observations. Since cross-validation folds share training observations, these were treated as repeated blocks rather than fully independent samples. *Post-hoc* pairwise comparisons were performed using the Nemenyi test when the overall test was significant.

#### 4.6.1 Comparison with baseline methods

Table 2 presents a comparison of the results achieved by the proposed method with baseline methods.

**Table 2:** Comparison of the proposed method with baseline methods.

Method	BuzzFeed	ISOT	Truthseeker
DT	67.1 ± 0.2	99.55 ± 0.1	62.3 ± 0.2
RF	66.53 ± 0.1	99.67 ± 0.1	70.1 ± 0.1
LR	60.98 ± 0.2	98.76 ± 0.2	63.1 ± 0.2
Proposed	85.21 ± 3.7	99.99 ±0.0	90.92 ± 0.1

As can be seen, the proposed method has achieved higher performance in comparison to all the baseline methods. An Iman-Davenport (Iman and Davenport, 1980) amended Friedman test (Friedman, 1937) was performed among the methods across the three datasets. A significant difference was found [*F*_*F*_(3, 42) = 49.00, *p* < 0.001], with the proposed method achieving the highest mean rank (1.00) across all datasets. Further, *post-hoc* pairwise comparisons using the Nemenyi test revealed that the proposed method performed significantly better than DT and LR (*p* < 0.01), as well as RF (*p* < 0.05).

#### 4.6.2 Comparison with state-of-the-art methods

Table 3 presents a comparison of the results achieved by the proposed method with state-of-the-art methods.

**Table 3:** Comparison of the proposed method with state-of-the-art methods.

Method	BuzzFeed	ISOT	Truthseeker
BERT (Devlin et al., 2019)	69.95 ± 0.1	99.33 ± 0.2	90.89 ± 0.2
Roberta (Liu et al., 2019)	75.47 ± 0.1	99.78 ± 0.1	90.44 ± 0.1
GPT2 (Brown et al., 2020)	70.42 ± 0.1	99.48 ± 0.2	89.79 ± 0.1
Proposed	85.21 ± 3.7	99.99 ± 0.0	90.92 ± 0.1

As can be seen, the proposed method achieved competitive performance across all three datasets. An Iman-Davenport amended Friedman test conducted among the four methods across the three datasets revealed a statistically significant difference in ranking, *F*_*F*_(3, 42) = 23.86, *p* < 0.001, with the proposed method achieving the highest mean rank (1.00) across all datasets. Further, *post-hoc* pairwise comparisons using the Nemenyi test revealed that the proposed method performed significantly better than BERT and GPT-2 (*p* < 0.01), while no significant difference was found between the proposed method and RoBERTa (*p* = 0.053).

Nevertheless, the proposed method significantly outperformed RoBERTa on the BuzzFeed dataset (*p* < 0.001). Moreover, the proposed method offers inherent explainability through its feature selection process. It provides interpretable insights into the features driving classification decisions. In contrast, the state-of-the-art methods (including RoBERTa) are black-box models that lack transparency in their decision-making processes.

#### 4.6.3 Comparison with similar studies

Table 4 presents a comparison of the results achieved by the proposed method with similar studies on fake news detection.

**Table 4:** Comparison of the proposed method with similar studies across different datasets.

Proposed method	Dataset	Similar studies
**85.21**	BuzzFeed	65.29 ↓ Buntain and Golbeck, 2017
		67.9 ↓ Yang et al., 2019
		86.0 → Choudhary and Arora, 2021
**99.99**	ISOT	99.65 → Rajalaxmi et al., 2022
		99.94 → Jiang et al., 2021
		98.89 ↓ Jain et al., 2025
**90.92**	TruthSeeker	89.0 ↓ Khalil and Azzeh, 2024
		96.10 ↑ Dadkhah et al., 2023
		92.8 → Arslan and Çelik, 2025

Bold values represent the accuracies achieved by the proposed method.

Table 4 employs the notation “ → ” and “↓” alongside the outcomes of comparative approaches to illustrate the relative performance of our proposed technique. The “↓” symbol denotes that the baseline method obtained significantly lower accuracy compared to our approach, while “ → ” signifies either no meaningful distinction or that the baseline method yielded comparable performance.

As can be seen, the proposed method achieved competitive results compared to similar studies carried out on the same datasets. Further, we performed a two-sample t-test with *Bonferroni correction* (α = 0.0167) and found that the proposed method (on average) achieved significantly higher performance than a number of the compared studies [*t*(44) = 2.93, *p* < 0.01; see Table 4], suggesting that it is both robust and well-suited for the task of fake news detection. These findings demonstrate the effectiveness and reliability of the proposed method in real-world scenarios.

#### 4.6.4 Interpretation of decision

The average number of features selected varied considerably across the three datasets, i.e., five for BuzzFeed, two for ISOT, and 59 for Truthseeker. This variation reflects the differing levels of complexity required to effectively distinguish between real and fake news within each source. Importantly, this selective approach improves feature-level interpretability by reducing the number of input variables required for decision-making and highlighting the most relevant discriminative cues.

For the BuzzFeed dataset, the best-performing fold highlighted features such as “unique words, secure domain usage, positive word frequency, word count, and text length.” This suggests that deceptive articles may rely on limited vocabulary, variable trust signals in links, and emotionally charged, shorter content. In contrast, ISOT's average of just two selected feature points to much simpler decision boundaries. Features like “subject and title length and subject with day” were consistently selected with high importance, indicating that minimal yet highly predictive metadata cues can effectively differentiate between real and fake news in this more structured dataset.

For the Truthseeker dataset, the top selected features were predominantly word2vec embeddings, supplemented by textual features such as the presence of hashtags, question count, and URLs. This indicates that fake news in the Truthseeker dataset tends to exploit a broader range of stylistic and structural strategies, making it necessary for the model to consider many complementary cues to capture its varied and often more subtle manipulative patterns. Overall, the proposed method's ability to select and focus on relevant features provides a more transparent representation of the decision process by highlighting the specific linguistic and structural cues contributing to classification.

#### 4.6.5 Comparison with SHAP and LIME interpretations

We compared the explanation of the PSO method with SHAP (Lundberg and Lee, 2017) and LIME (Ribeiro et al., 2016) on the BuzzFeed dataset.

The PSO-based feature selection method identified source domain as the most consistently selected feature (across 4 of 5 folds), followed by the presence of exclamation marks and negative words (2 folds each), and has questions, title length, and unique words (1 fold each). These results suggest that the PSO method prioritizes linguistically and source-based discriminative features, with source domain emerging as the most stable and informative predictor of news authenticity across cross-validation folds. The variability in other features across folds further indicates that PSO adaptively identifies context-sensitive features, rather than relying on a fixed feature set.

Figure 2 illustrates the global feature importance rankings derived from SHAP. Among the top-ranked features are source domain, has quotes, title length, positive words, has exclamation, unique words, and text length. This suggests that, at a global level, the model's predictions are predominantly driven by source credibility signals and stylistic linguistic cues, consistent with prior literature indicating that writing style and source origin are reliable indicators of news veracity (Heuer and Glassman, 2024).

**Figure 2:**
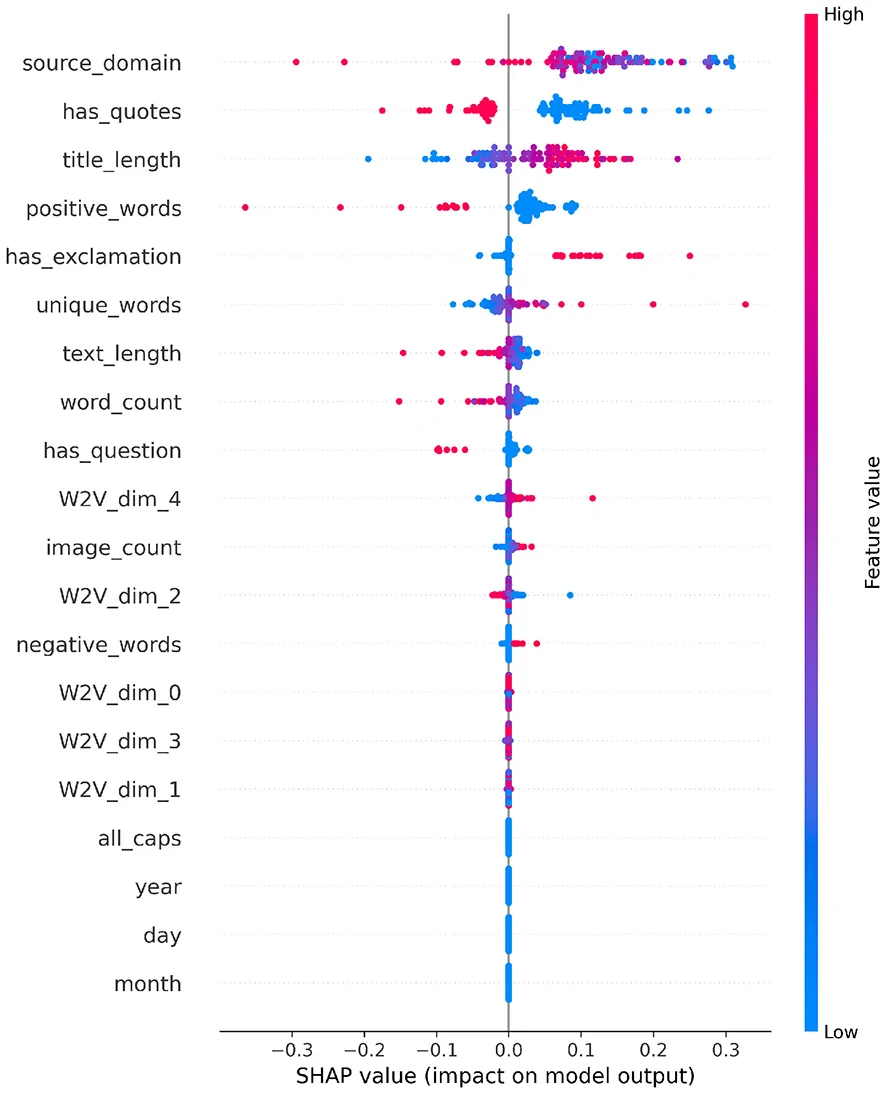
Global feature importance using SHAP values for the trained classifier.

Similarly, Figure 3 illustrates the LIME explanation for a selected instance of a true news article. The green bars represent features that contribute positively toward classifying the instance as true news, while the red bars represent features that work against this classification, i.e., features whose values in this instance are more characteristic of fake news and thus reduce the model's confidence in predicting the instance as true. Specifically, when high values of 'has exclamation, negative words, and image count' were observed, these features individually reduced the likelihood of classifying the article as real news, whereas source domain, has questions, and unique words contributed positively to the true news prediction.

**Figure 3:**
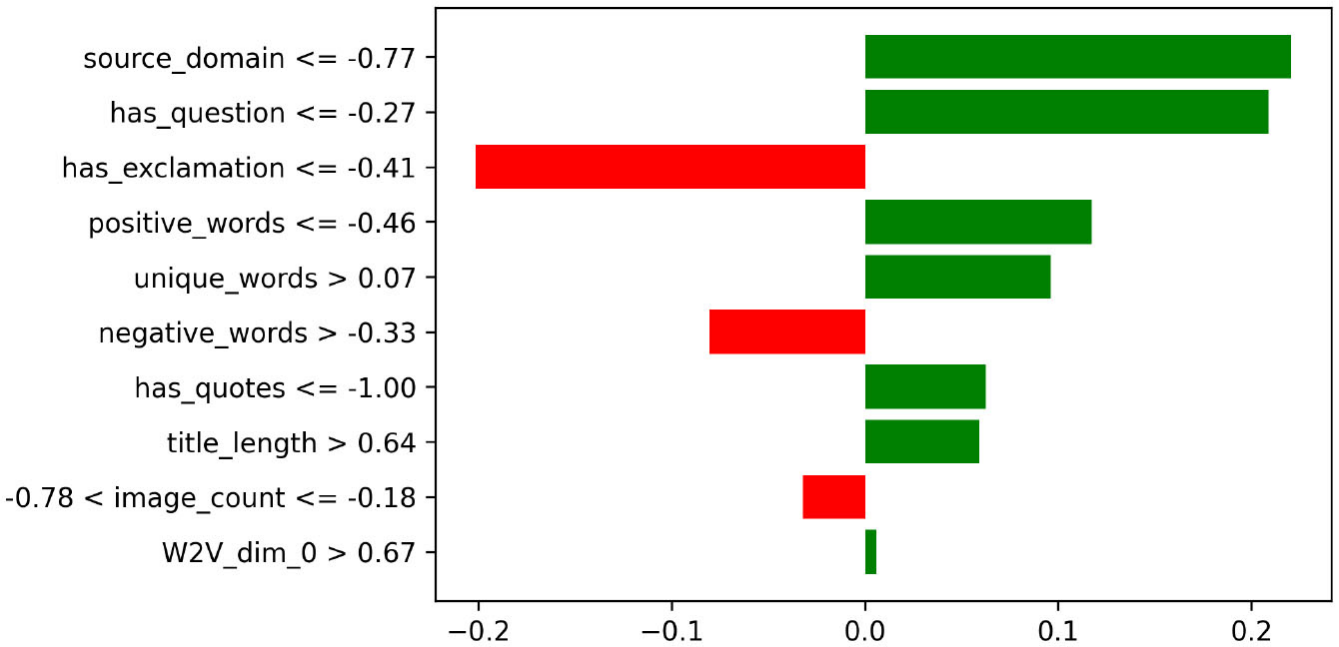
Local explanation of an individual prediction using LIME. Red bars indicate features against the prediction, whereas green bars indicate features in favor of the prediction. Note that this instance is classified as real news with a probability of 75%.

Taken together, the features identified by PSO show notable overlap with those highlighted by both SHAP at the global level and LIME at the local level, particularly source domain, unique words, and stylistic features. This suggests convergent validity across the three methods. However, while SHAP and LIME serve as *post-hoc* explainability tools that operate on an already-trained model, the PSO-based method functions as an embedded feature selection mechanism that directly shapes the model's input space prior to training. PSO reduces feature dimensionality and potential noise before learning, which may contribute to improved generalization, whereas SHAP and LIME provide complementary interpretability of the trained model's behavior. The consistency in features identified across all three methods strengthens confidence in the relevance of the selected feature subset and provides additional evidence that the selected features capture meaningful predictive patterns.

#### 4.6.6 Computational efficiency

The proposed method demonstrated clear advantages in computational efficiency across all three datasets when compared to state-of-the-art transformer-based models.

For the BuzzFeed dataset, the proposed method completed its run in less than 1 min, outperforming BERT (1 min 6 s), GPT (1 min 10 s), and RoBERTa (1 min 4 s). While all models were reasonably fast on this smaller dataset, the proposed approach still showed a modest time advantage.

The difference became much more pronounced on the ISOT dataset. The proposed method finished in under 30 min, whereas GPT took 5.4 h, RoBERTa 4.9 h, and BERT 6.7 h. This substantial reduction in runtime highlights the efficiency of the proposed approach when handling larger or more complex data.

For the TruthSeeker dataset, the proposed method completed its processing in about 35 min, significantly faster than BERT (> 11 h), RoBERTa (> 12 h), and GPT (> 14 h). This result emphasizes the scalability and practicality of the proposed method for time-sensitive or resource-constrained settings, making it a strong alternative to existing state-of-the-art models.

## 5 Discussion

This paper proposed an explainable approach to fake news classification using Particle Swarm Optimization (PSO). The proposed method leverages Mutual Information (MI) in its fitness function to guide the feature selection process, thereby improving classification performance by eliminating redundant or irrelevant features. The proposed method achieved accuracies of 85.21% on the Buzzfeed dataset, 99.99% on the ISOT dataset, and 90.92% on the TruthSeeker dataset. These results demonstrate its effectiveness across multiple domains and sources, highlighting its potential as a generalizable approach to fake news detection.

Further, the method achieved an average accuracy of 92.04% across all datasets, outperforming baseline methods (which all achieved less than 79% accuracy) and showing competitive results compared to state-of-the-art models such as BERT (86.22%), RoBERTa (88.56%), and GPT-2 (86.56%). Although the accuracy improvement over these state-of-the-art methods is not statistically significant, the proposed method delivers comparable performance while requiring significantly less computational time, making it an attractive option for scenarios with limited computational resources.

We have also investigated the effectiveness of the proposed method against recent instruction-based models, specifically GPT-5 (see Supplementary material for a detailed description of the GPT-5 experimental setup). We found that the proposed method achieved higher accuracy than GPT-5 on two datasets (ISOT: 99.99% vs. 59.25% and TruthSeeker: 90.92% vs. 65.67%), with the exception of the BuzzFeed (BuzzFeed: 85.21% vs. 86.26%). This demonstrates that the proposed approach remains highly competitive even when compared with advanced instruction-based models. The strong performance can be attributed to the task-specific feature selection mechanism, which identifies the most informative features for fake news detection, while instruction-based models rely on general-purpose language understanding capabilities.

Most importantly, the method offers enhanced explainability. For instance, the features selected by PSO with MI provide clear, interpretable insights into the classification decision process. While transformer-based models are somewhat explainable through their attention weights, these weights can be diffuse, hard to interpret consistently, and do not directly identify the most relevant input features in the context of fake news detection. By contrast, the proposed method using PSO explicitly selects and ranks meaningful features, offering clearer and more actionable explanations for its predictions. The selected features have been shown to improve transparency and facilitate human understanding of the factors driving the detection of fake news. It is also noted that the extracted sentiment features, which we added to the TruthSeeker dataset, were selected as important predictors by the method. This validates our hypothesis that sentiment cues are informative for distinguishing real from fake news, suggesting that emotional tone and polarity can serve as meaningful signals in automated disinformation detection systems.

Furthermore, the additional stability analysis demonstrated that the proposed PSO-based feature selection procedure was highly reproducible under a fixed random seed. PSO runs within each of the five outer folds resulted in identical feature subsets across all repeated runs. Accordingly, both the mean Jaccard similarity and Kuncheva stability index were 1.00 (SD = 0.00). This indicates that, when the stochastic conditions of the optimization are controlled, the proposed method consistently converges to the same feature subset within each fold. Across the 100 PSO runs, an average of 5.00 features (SD = 1.68) were selected. The source_domain feature was selected in all runs (100%), while text_length and word_count were selected in 80% of the runs, and the has_quotes feature was selected in 40% of the runs. The corresponding mean test accuracy was 86.12% (SD = 4.61), ranging from 75.00% to 89.19% across the five outer folds. These results demonstrate consistent predictive performance on unseen test data while producing identical feature subsets across repeated runs under the fixed random seed.

To further analyse the computational cost of the proposed framework, we decomposed the total execution time into three components: feature extraction, training, and testing. For the proposed PSO-based method, the training stage includes both the PSO-based feature selection process and the subsequent classifier training. Across all datasets, feature extraction accounted for only a small proportion of the total runtime (approximately 1–5%), while the testing phase consistently represented less than 1% of the overall execution time. For the BuzzFeed dataset, feature extraction time was negligible due to the relatively small dataset size, resulting in a negligible contribution to the overall runtime. In contrast, feature extraction accounted for approximately 5% of the runtime for the ISOT and TruthSeeker datasets because of their larger sample sizes and higher feature dimensionality. Consequently, the majority of the computational cost was attributed to the training stage for both the proposed method and the state-of-the-art methods. Nevertheless, despite including the additional PSO-based feature selection process, the total training time of the proposed framework remained substantially lower than that of the transformer-based state-of-the-art models, demonstrating that the computational overhead introduced by PSO is modest relative to the overall training process while still providing a significant reduction in training time (see Section 4.6.6).

It is also worth noting that the features selected by the proposed method align closely with those identified by *post-hoc* explanation methods such as SHAP and LIME, as demonstrated in Section 4.6.5. This convergence further validates the relevance and reliability of the selected feature subset. Nevertheless, the proposed method holds a distinct advantage in that feature selection is performed prior to training, thereby reducing the dimensionality of the input space, lowering computational overhead, and mitigating the risk of overfitting caused by irrelevant or redundant features. These are benefits that *post-hoc* methods, which operate on an already-trained model, do not provide.

Beyond the binary classification tasks evaluated throughout this study, we further assessed the generalizability of the proposed framework on the six-class PolitiFact dataset, comprising the classes true, mostly true, half-true, mostly false, false, and pants on fire. The proposed approach achieved an accuracy of 34%, outperforming the state-of-the-art methods, which reported accuracies below 27%. Although the overall accuracy is lower than that obtained on binary classification datasets due to the increased complexity of fine-grained veracity prediction, these results demonstrate that the proposed method remains effective and generalizes well to more challenging multi-class fake news classification tasks.

The use of MI in the fitness function was inspired by adaptive sensing techniques in previous studies (Morssy et al., 2023), which demonstrated improved performance and enhanced explainability in image-based tasks. To further examine the impact of MI in our context, we adapted the same approach to work with disinformation detection on the Buzzfeed dataset using only the features selected by PSO without MI. Interestingly, this adaptive sensing variant categorized the real/fake classes primarily using the “datetime” feature. This suggests that the posting date and time may carry valuable signals about content authenticity. This finding is supported by Vargas et al. (2020), who also found that coordinated campaigns often show suspicious temporal patterns. However, this approach achieved only 75.68% accuracy, significantly lower than the 85.21% achieved by the proposed method. Future work should therefore aim to enhance the adaptive sensing approach by incorporating richer textual features and developing domain-specific representations that better capture linguistic cues relevant to disinformation. This could improve classification performance while further reducing computational cost.

Overall, the proposed method offers a practical, explainable, and computationally efficient solution for fake news detection. Its ability to deliver competitive accuracy with clear explanations at the feature level makes it valuable for high-stakes settings where transparency is critical, such as journalism, public policy, and the moderation of social networks.

However, this study has several limitations. First, the current evaluation was limited to an in-distribution setting, where the training and test samples were drawn from the same dataset. Consequently, further investigation is required to assess the generalizability of the proposed method under cross-dataset and out-of-distribution conditions. In particular, temporally separated splits and cross-source validation would provide stronger evidence of the model's ability to adapt to evolving misinformation patterns and unseen sources.

Second, while the selected features provide evidence of feature-level interpretability, a comprehensive evaluation of explainability would require additional user-centered studies and quantitative fidelity assessments. Future work could investigate whether domain experts can effectively use the generated explanations for auditing decisions or evaluate explanation fidelity using metrics such as sufficiency, comprehensiveness, or perturbation-based measures.

Third, although the proposed method demonstrates competitive performance on benchmark datasets, its robustness and fairness under real-world conditions require further examination. The selected features may be influenced by dataset-specific characteristics, such as source-domain patterns, which could introduce potential bias and limit generalization. Additionally, the resilience of the proposed method against adverse perturbations and evolving misinformation strategies has not been explicitly evaluated. Future research should address these aspects through dedicated robustness and bias analyses, including adversarial testing, fairness assessment, and evaluation on real-time news streams, diverse languages, and different cultural contexts. These investigations would provide deeper insights into the reliability, practical deployment potential, and broader applicability of the proposed explainable hybrid approach to fake news detection.

## 6 Conclusion

This paper proposed an explainable approach to fake news detection by combining Particle Swarm Optimization (PSO) with Mutual Information (MI) for feature selection. The method demonstrated strong performance across multiple datasets, achieving an average accuracy of 92.04%, while outperforming baseline models and performing competitively with state-of-the-art models such as BERT, RoBERTa, and GPT-2. In addition to its high accuracy, the method offers a significant advantage in computational efficiency and interpretability, making it suitable for practical deployment in real-world applications. The selected features provide meaningful explanations for the classification outcomes, addressing a key limitation of black-box models. Future work could explore integrating domain knowledge or user feedback into the feature selection process to further enhance interpretability and tailor the model to specific disinformation contexts or evolving narratives.

## Data Availability

The original contributions presented in the study are included in the article/Supplementary material, further inquiries can be directed to the corresponding author.
