# Supplementary material for: An explainable approach to fake news detection using PSO and mutual information
Source: Front Artif Intell. 2026 Oct 7;9:1901946. doi: 10.3389/frai.2026.1901946 (PMC13683683; doi:10.3389/frai.2026.1901946)
Supplement: Supplementary file 1 [file Supplementary_file_1.pdf]

Supplementary Section

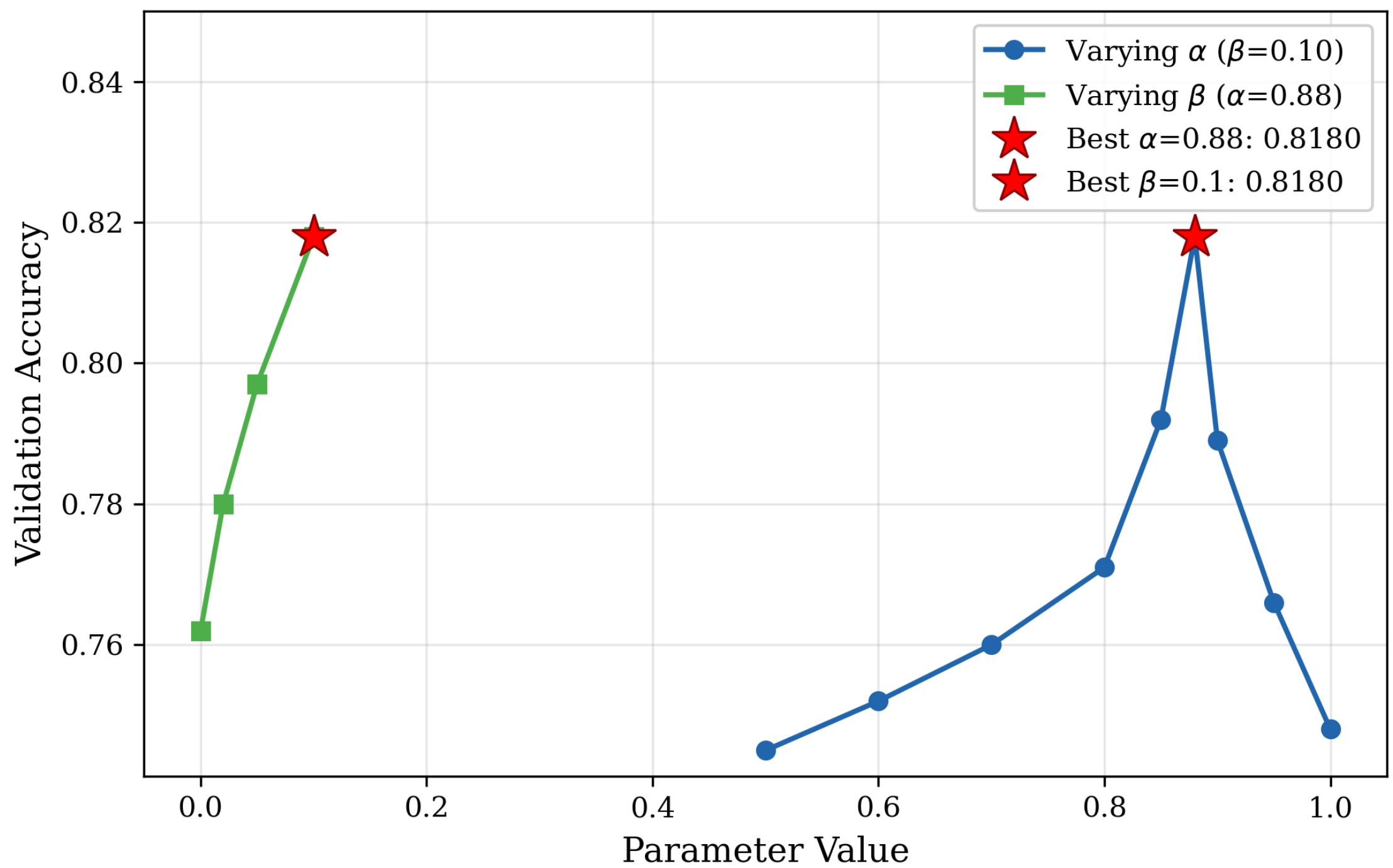

Figure A.1: Sensitivity analysis showing the effect of varying α (weighting the fitness function) and β (weighting mutual information).

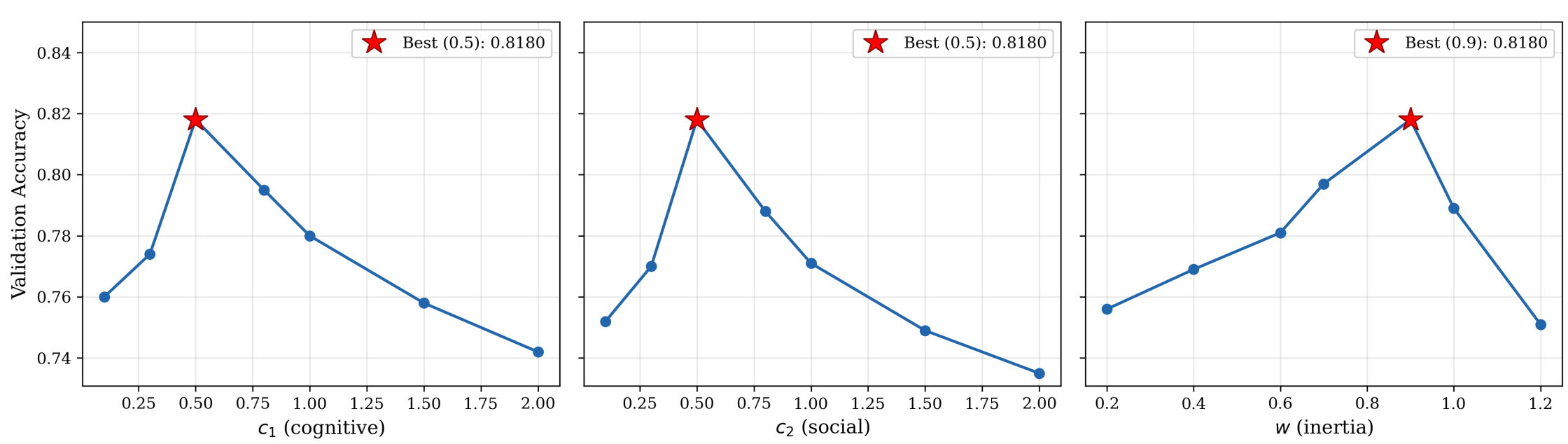

Figure A.2: Sensitivity analysis showing the effect of varying the PSO parameters: inertia weight (w), cognitive coefficient (c_1), and social coefficient (c_2)

.

0.1. GPT-5 Experimental Protocol

For the comparison with a large language model, GPT-5 was evaluated on all three datasets using a zero-shot prompting protocol. To manage the associated API usage costs, 10,000 stratified randomly selected examples were evaluated from the ISOT and TruthSeeker datasets, while all 182 instances from the BuzzFeed dataset were used. GPT-5 was accessed through the OpenAI API on 25 July 2026 using

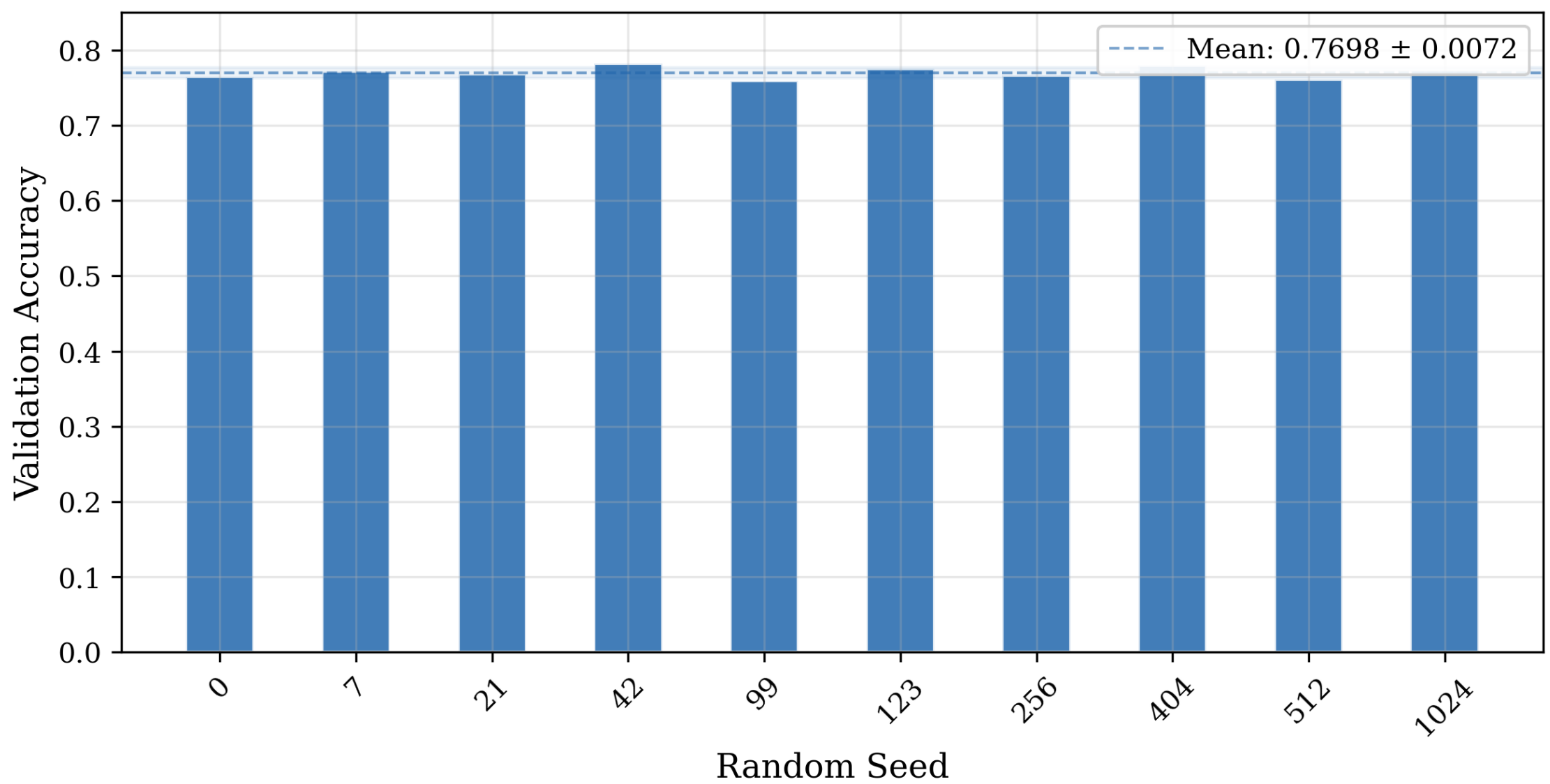

Figure A.3: Sensitivity analysis showing the effect of varying the random seed values.

the following parameters: `model = gpt-5`, `temperature = 0.0`, `max_tokens = 10`, and an input truncation length of 8,000 characters. The following prompt was used:

> *You are an expert fact-checker. Analyze the following article text and determine if it is likely to be real (factual) or fake (misleading/false). Consider the language used, claims made, and overall credibility. Respond with only 'real' or 'fake'.*

For the evaluation, the dataset was partitioned into five folds using shuffled K-fold cross-validation with a fixed random seed of 42. GPT-5 was independently evaluated on the articles in each held-out fold; the remaining folds were not used to train or fine-tune the model. The model output was mapped to the two classes, with "real" assigned to the *real* class and "fake" assigned to the *fake* class. The resulting predictions were then compared with the ground-truth labels to calculate classification accuracy.
